# Supplementary material for: Plasmid-borne mcr-1 and replicative transposition of episomal and chromosomal blaNDM-1, blaOXA-69, and blaOXA-23 carbapenemases in a clinical Acinetobacter baumannii isolate
Source: mSystems. 2025 Feb 24;10(3):e01683-24. doi: 10.1128/msystems.01683-24 (PMC11915867; doi:10.1128/msystems.01683-24)
Supplement: Supplemental Figures — Figures S1 to S9. [file msystems.01683-24-s0005.pdf]

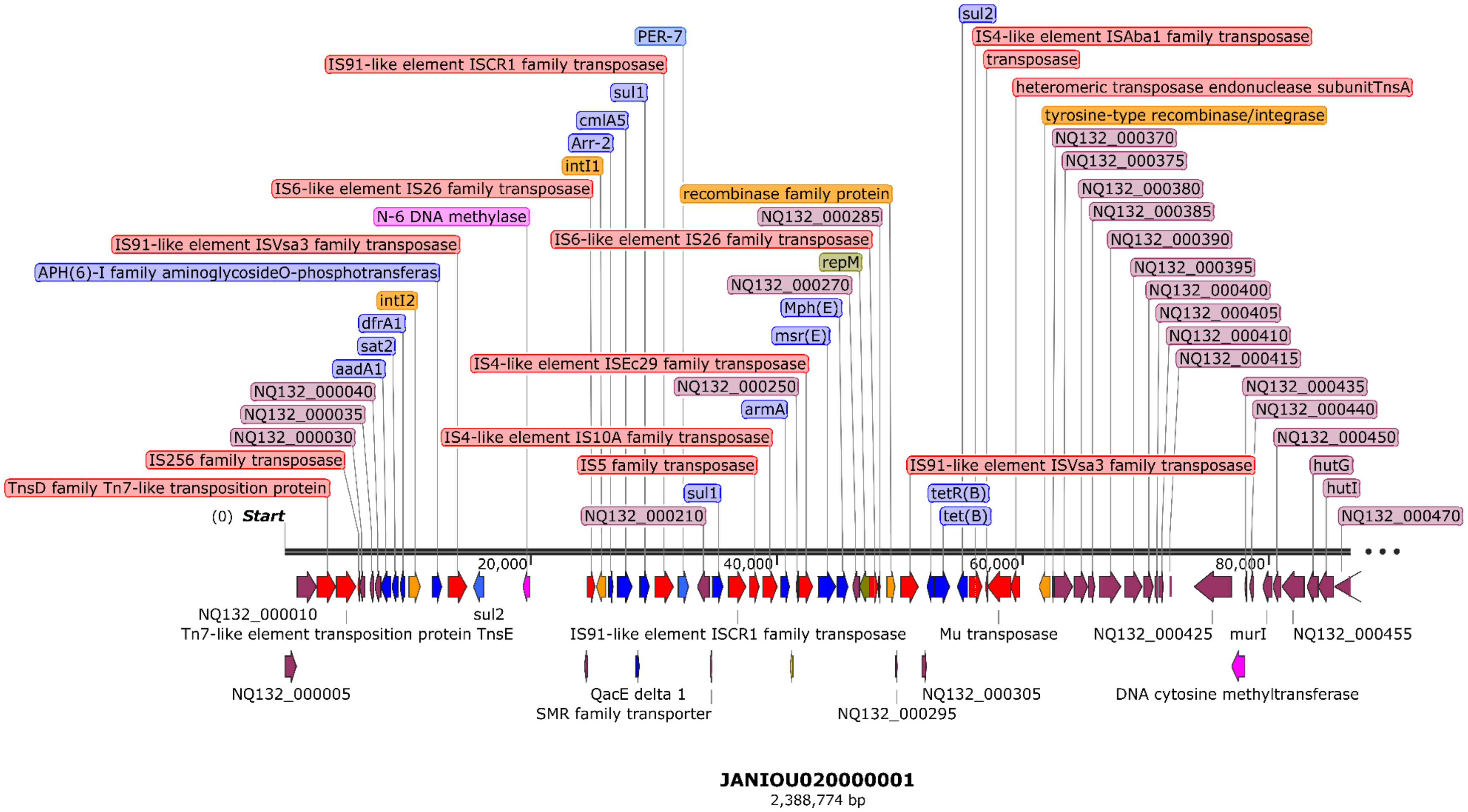

***Figure S1. Genetic environment of resistance genes found on a genomic island on a chromosome (contig 1: 0kb to 85kb).** All the resistance genes (shown in blue) were bracketed by mobile genetic elements (shown in red and orange) and clustered together on the island. The close alignment of this region with other plasmids and chromosomes show that this region was a plasmid that was integrated into the chromosome. Epigenomically, DNA methyltransferases and methylase were also found within the genomic island.*

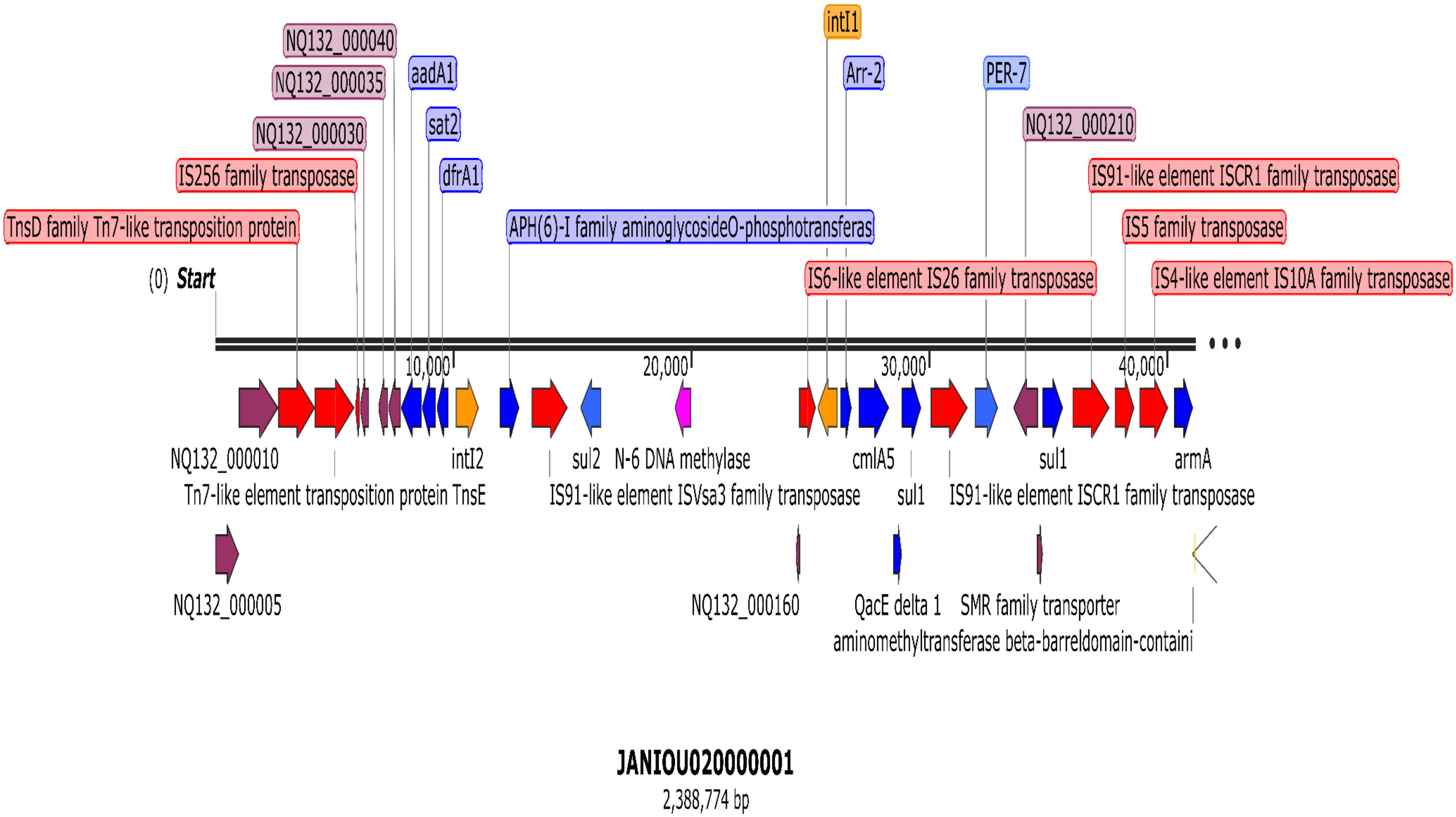

Figure S2. *Genetic environment of resistance genes found on contig 1 (between 0kb to 40kb) shows mobile genetic elements bracketing the resistance genes. This image is an expansion of Figure S1 between the 0 - 40kb region above.*

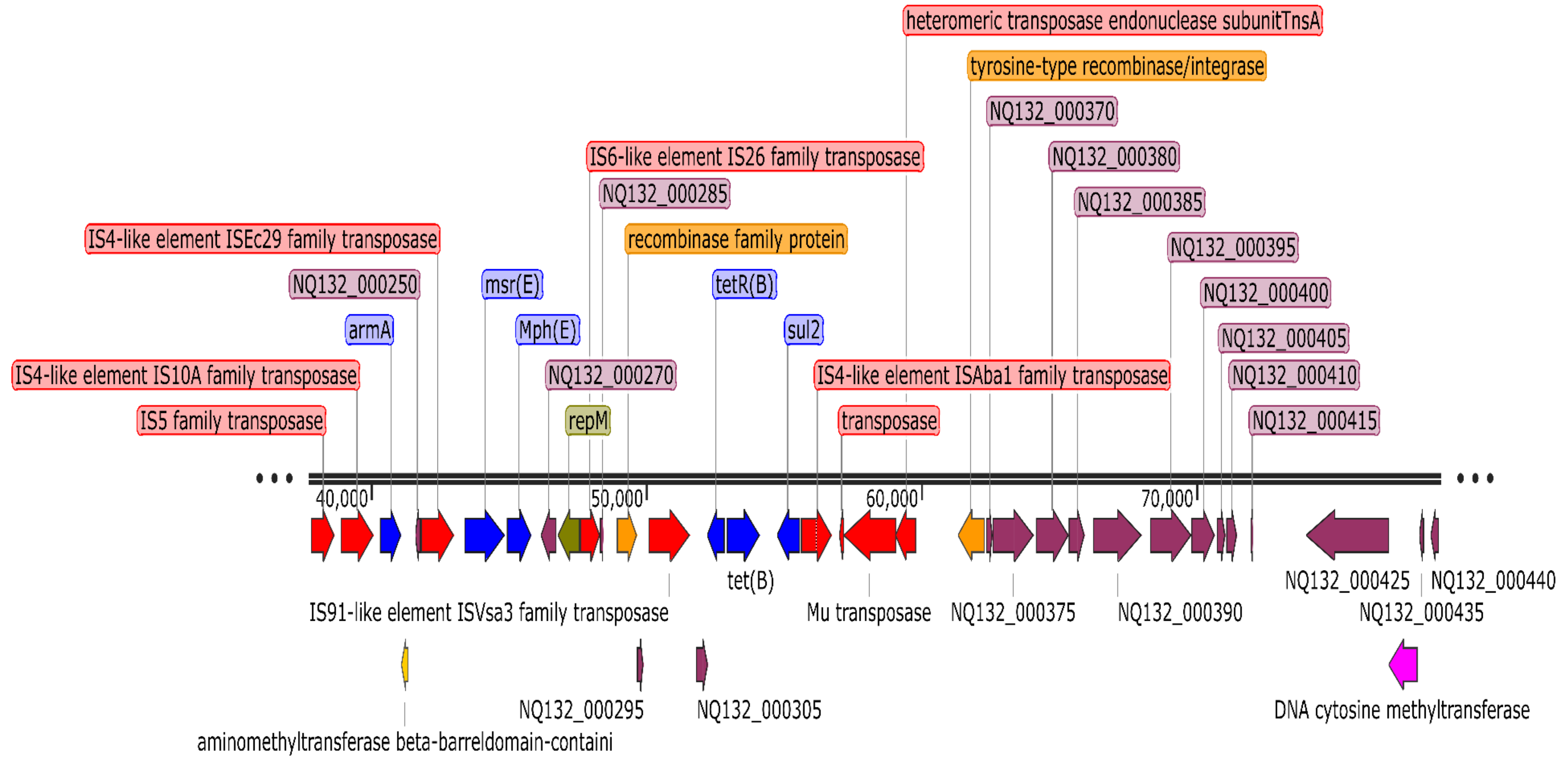

Figure S3. *Genetic environment of resistance genes found on contig 1(between 40kb to 80kb) shows mobile genetic elements bracketing the resistance genes. This image is an expansion of Figure S1 between the 40 - 80kb region above.*

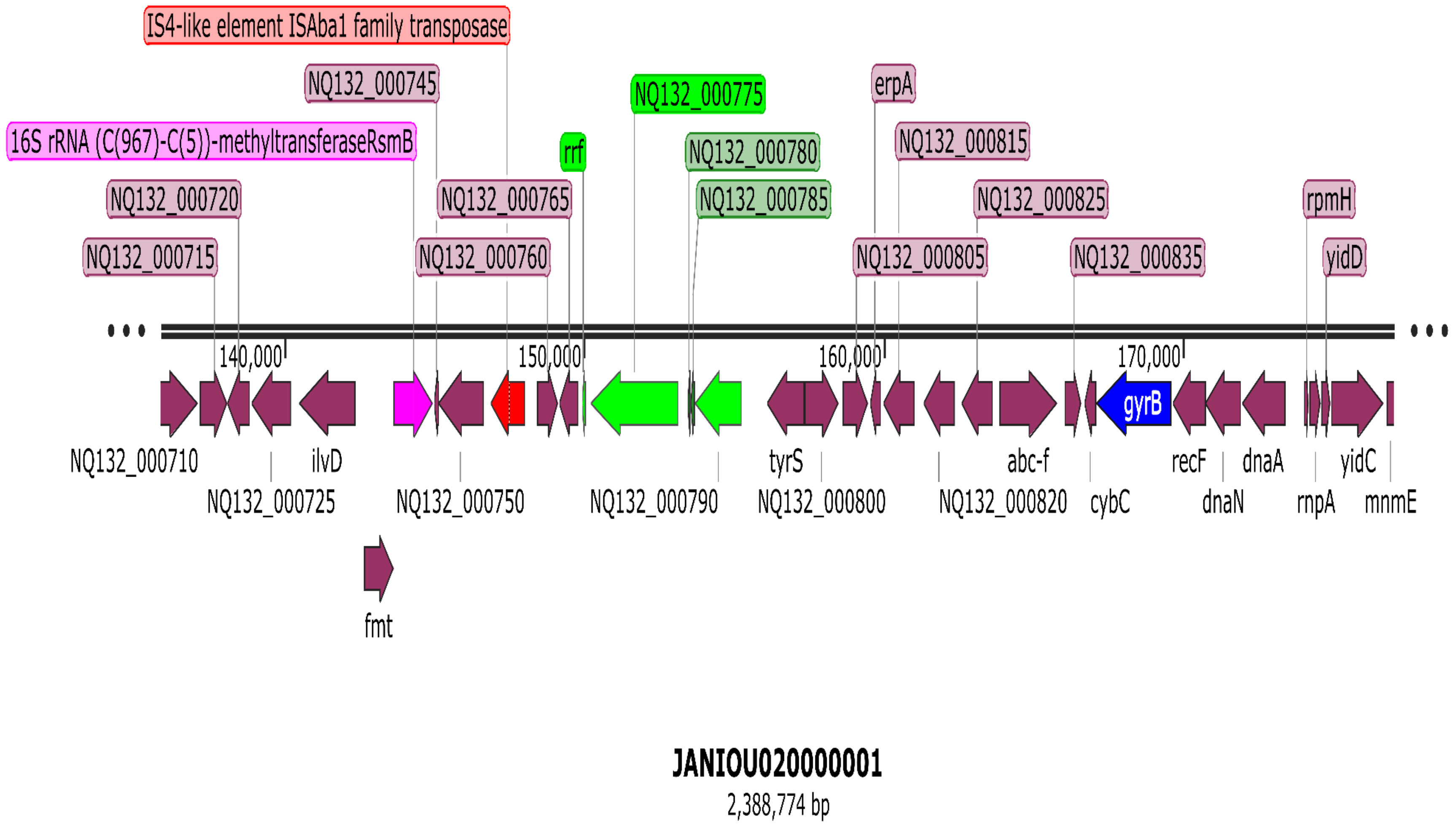

Figure S4 Genetic environment of resistance genes found on contig 1 (~135kb-180kb) shows mobile genetic elements bracketing the resistance genes. *This image is based on the region between 130kb and180kb, showing a methyltransferase to epigenetic gene regulation. gyrB, which can confer resistance to fluoroquinolones when there are mutations, was found in this region without any MGE around it.*

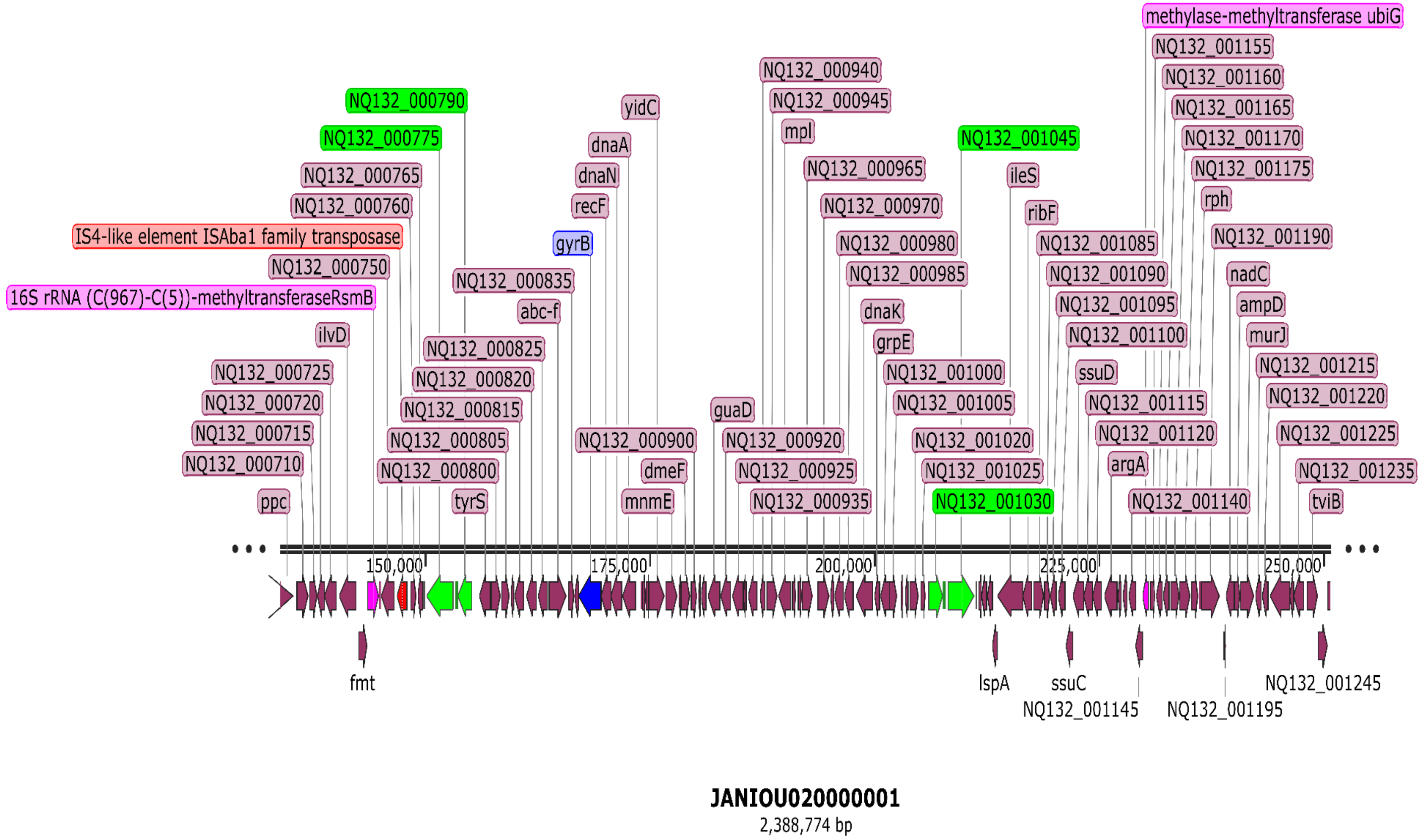

***Figure S5. Mobile genetic elements and methyltransferases on contig 1 between 120kb to 250kb region**. This region harbours methyltransferases, gyrB, and an ISAba1 mobile genetic element.*

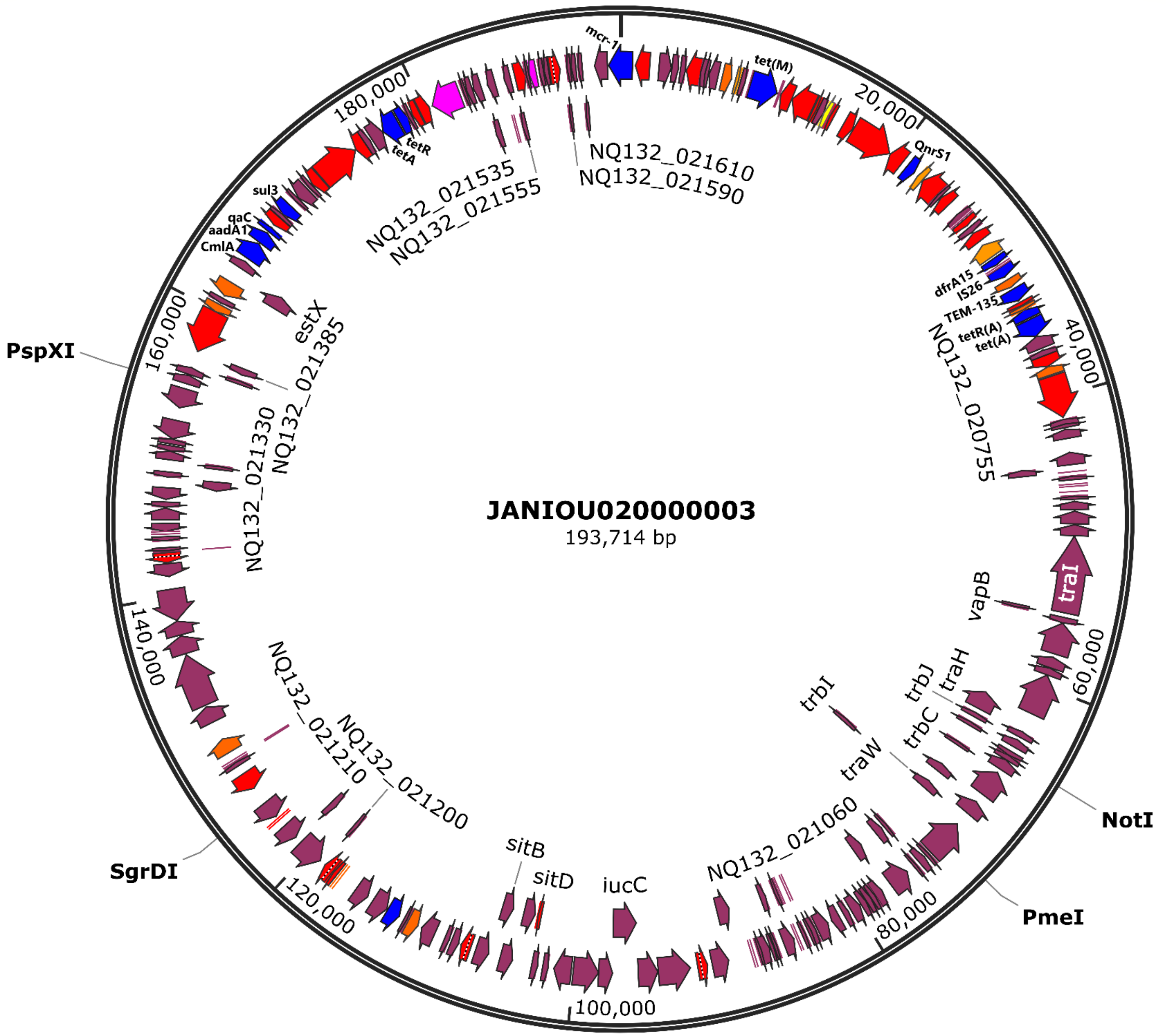

***Figure S6. Genetic environment of resistance genes found on plasmid pR-B2.MM_C3**. The plasmid contains methyltransferases, composite transposons, and integrons bracketing the resistance genes, which were clustered together within a genomic island surrounded by mobile genetic elements such as integrons, composite transposons, and insertion sequences.*

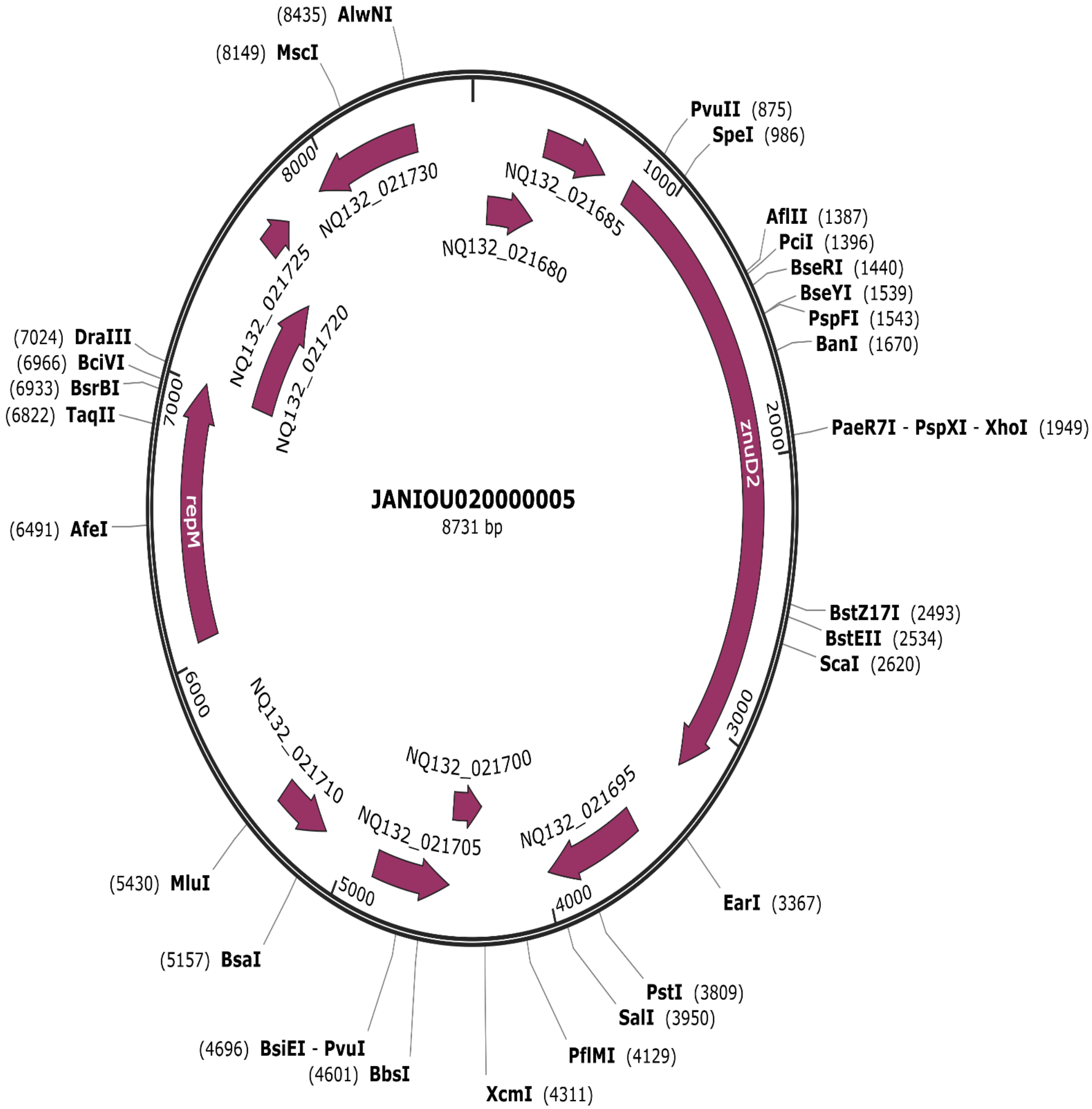

Figure S7. Genetic map of pR-B2.MM_C6. *A circularized map of pR-B2.MM_C6. pR-B2.MM_C6 has no resistance gene.*

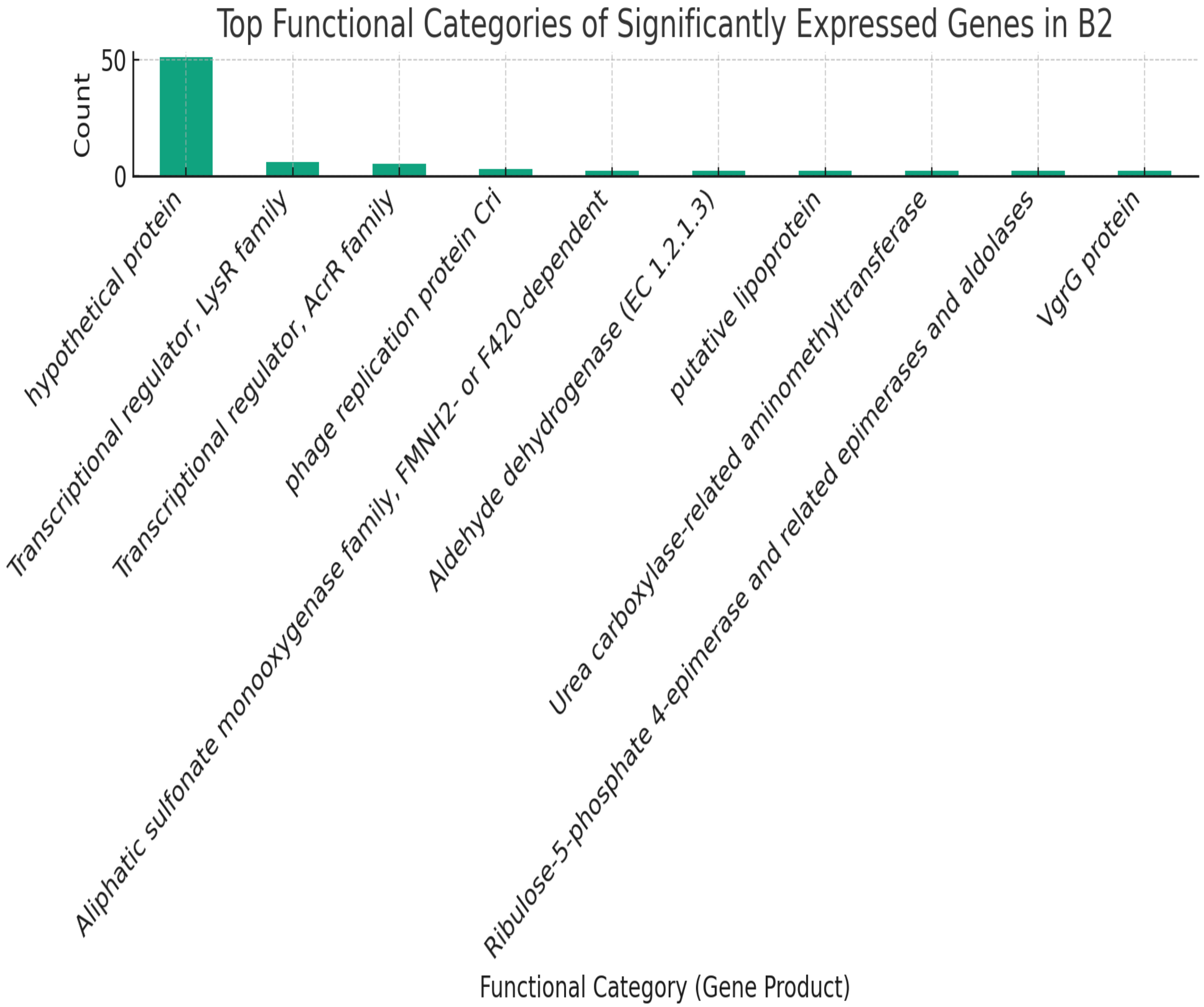

Figure S8. Top functional categories of significantly and differentially expressed genes (DEGs) in isolate R-B2. *Most of the DEGs belonged to hypothetical proteins with no known function, transcriptional regulators, and mobile genetic elements.*

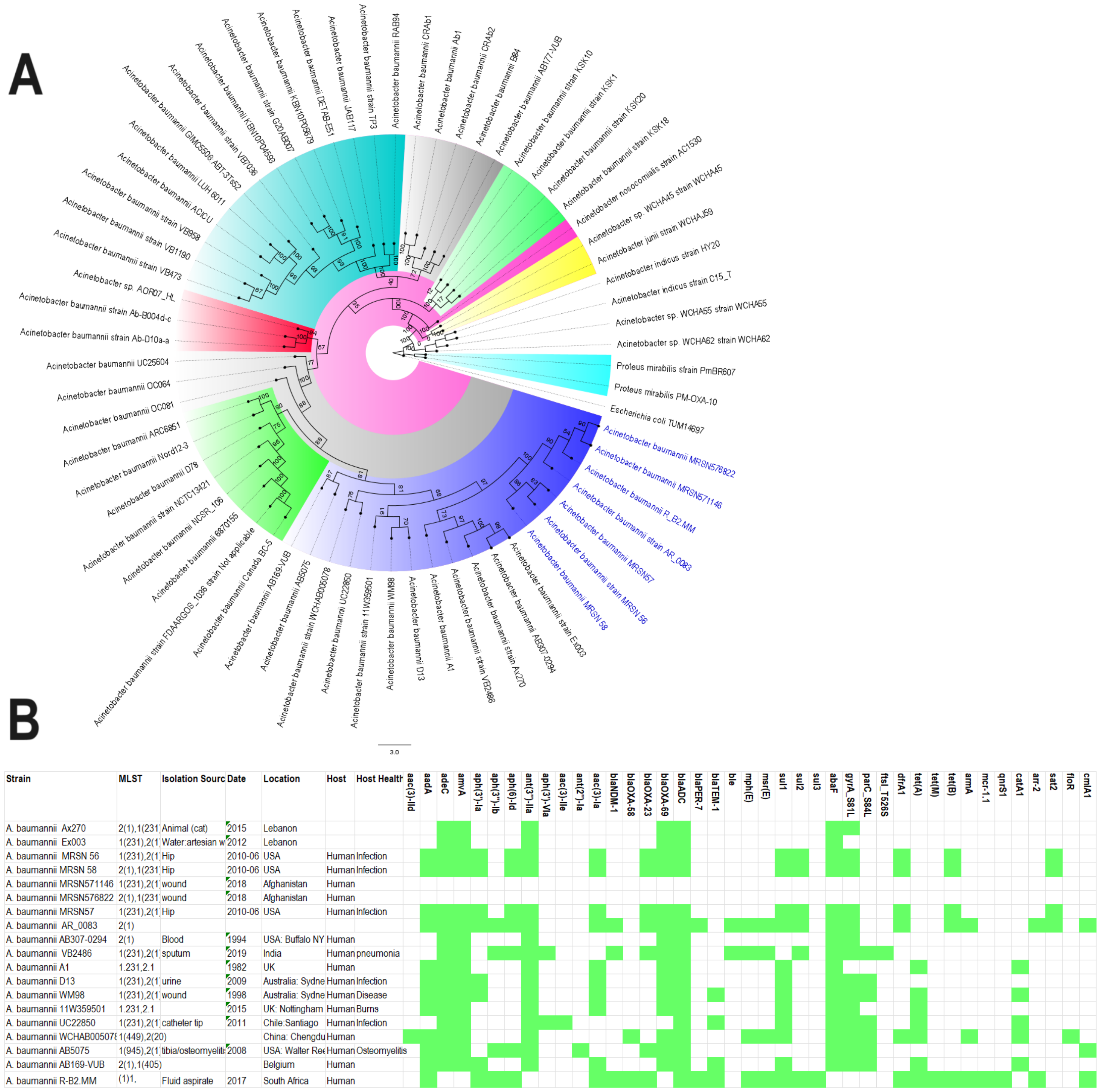

Figure S9. Phylogenetic analysis of antibiotic-resistant global Acinetobacter baumannii strains with close sequence homology to R-B2.MM. *A. baumannii strains that had close sequence homology to R-B2.MM after nucleotide BLAST showed very close evolutionary association with the R-B2.MM. The strains with very close evolutionary association to the R-B2.MM strain are shown as blue text in A. The resistome is shown in B. The resistome were not conserved across the strains on the same clade. Resistance genes that were conserved across the species includes the bla*$_{OXA\text{-}69}$ and *bla*$_{ADC}$.

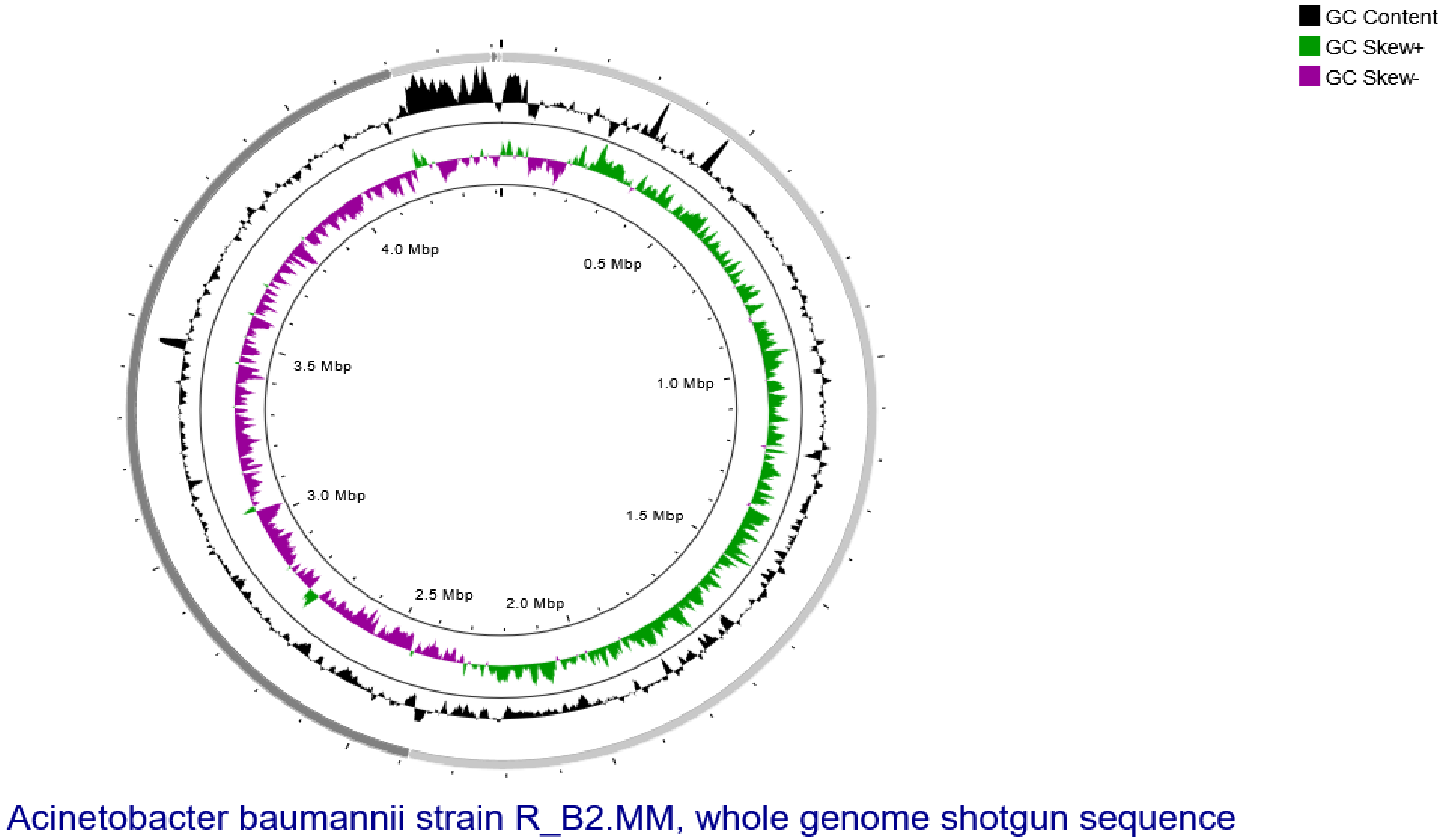

Figure S10. Genome map of R_B2.MM strain with GC content annotation

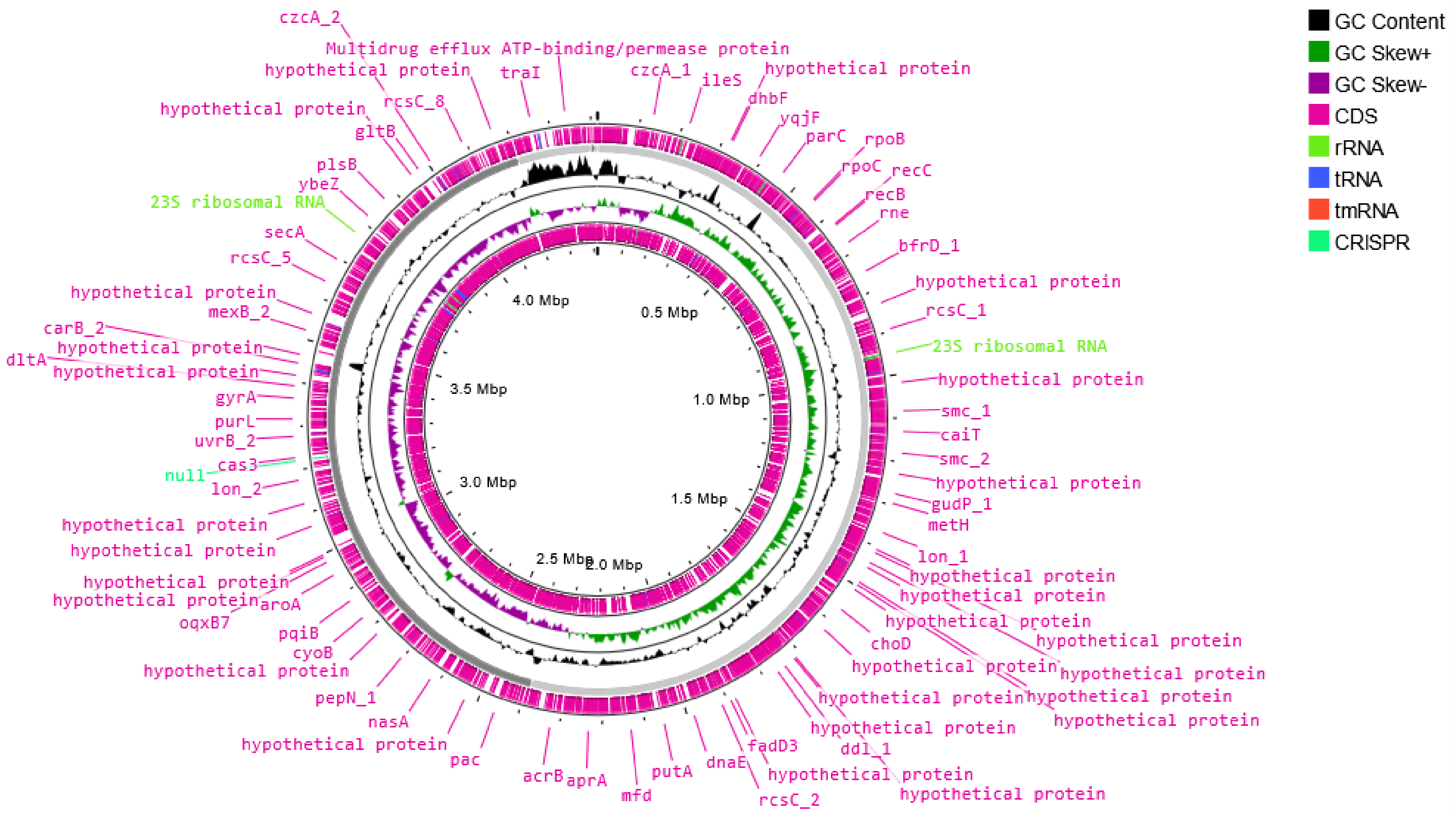

Acinetobacter baumannii strain R_B2.MM, whole genome shotgun sequence

Figure S11. Prokka annotation of the coding sequences, RNAs, and CRISPR elements in the genome map of A. baumannii R_B2.MM

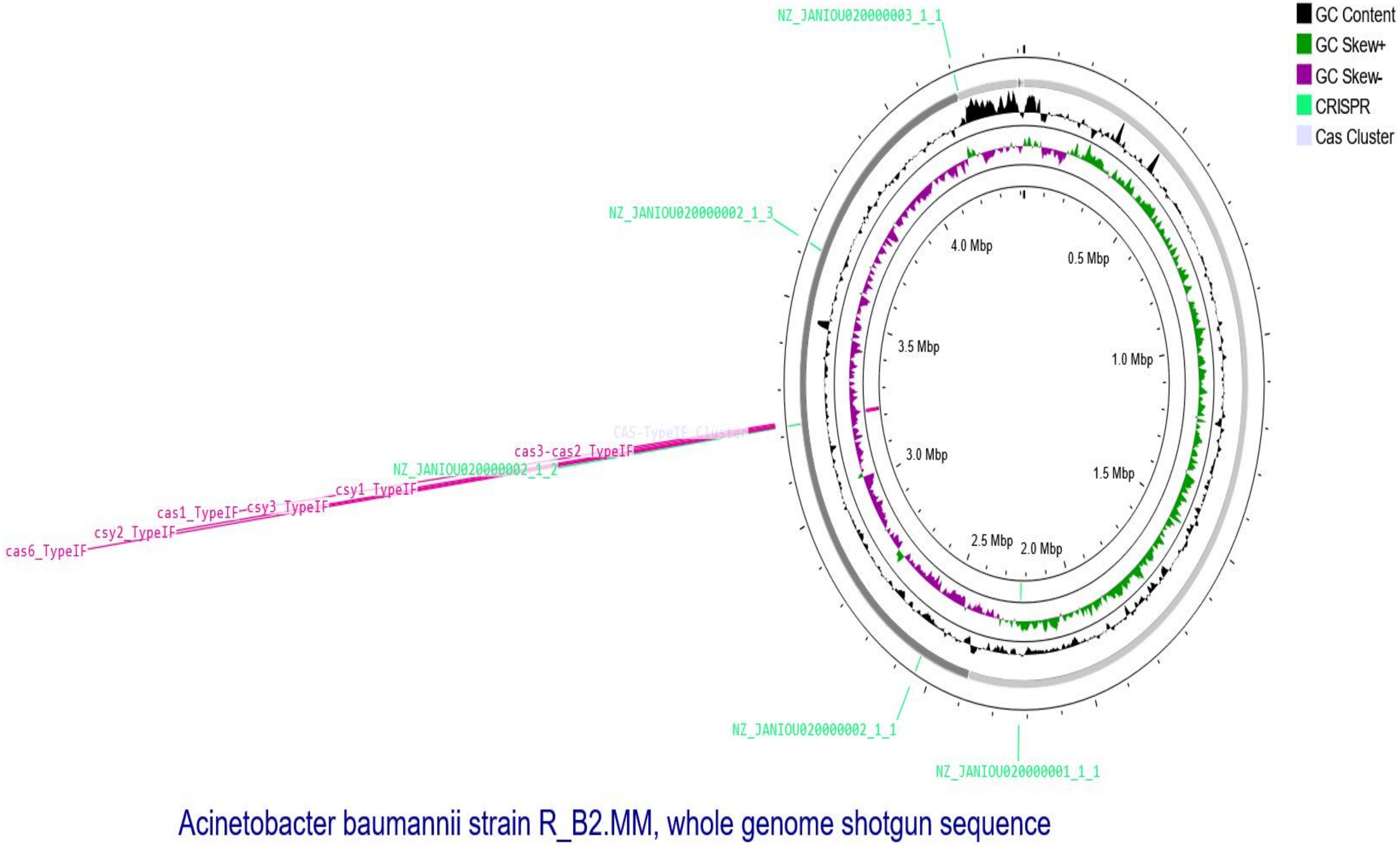

Figure S12. CRISPR systems and Cas proteins found in the genome of A. baumannii R_B2.MM

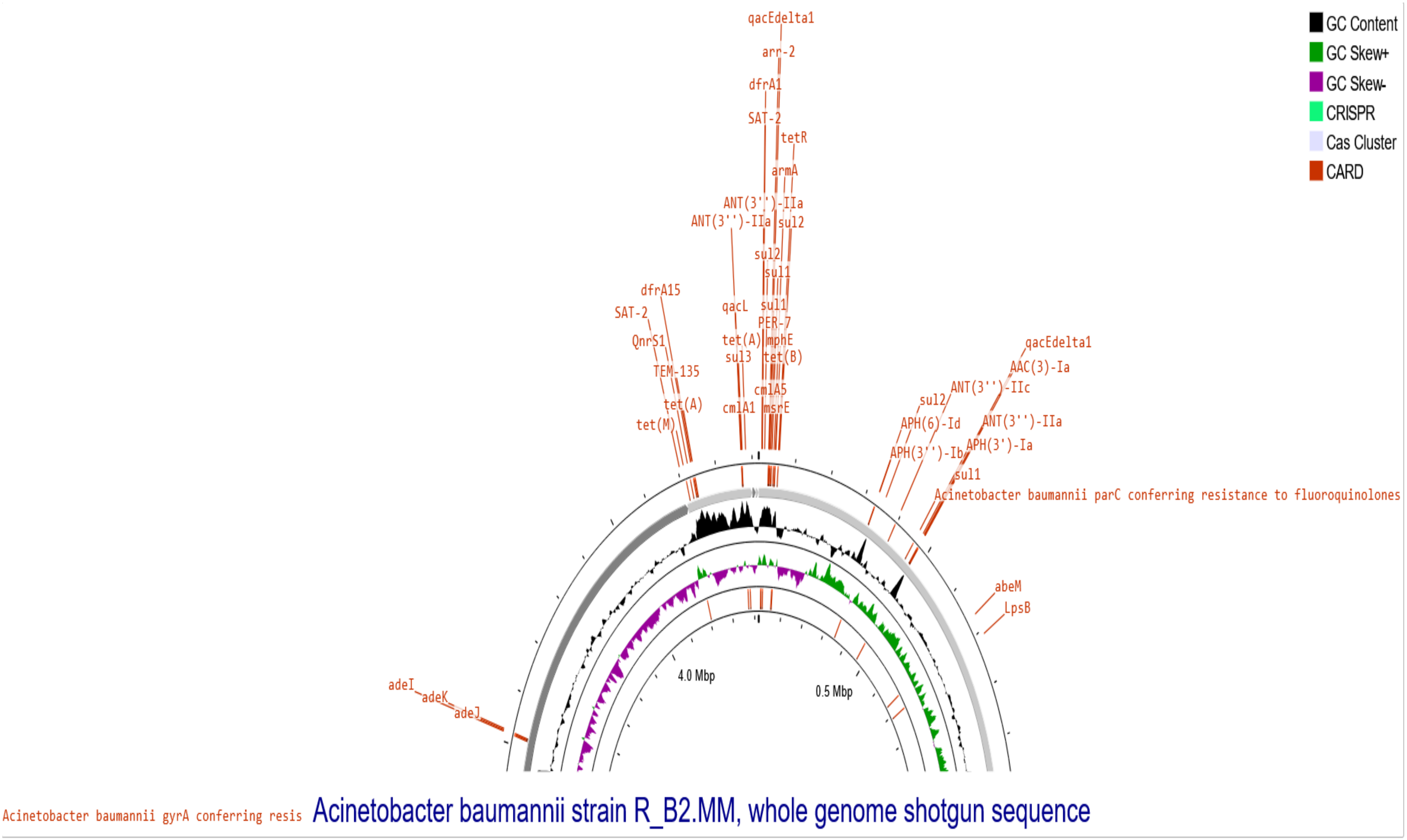

Figure S13a. CARD-annotated resistance genes found in the genome of A. baumannii R_B2.MM strain.

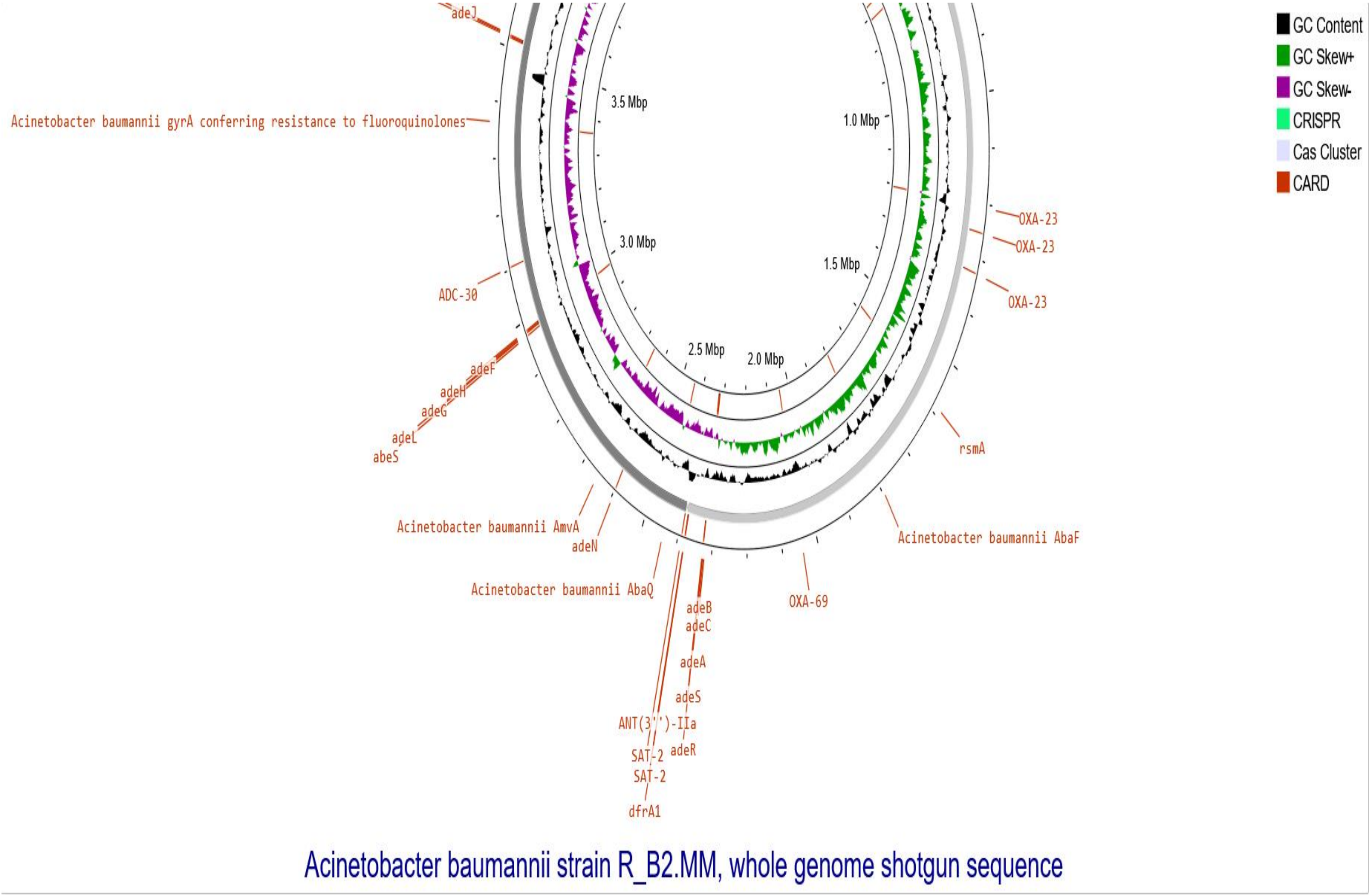

Figure S13b. CARD-annotated resistance genes found in the genome of A. baumannii R_B2.MM strain.

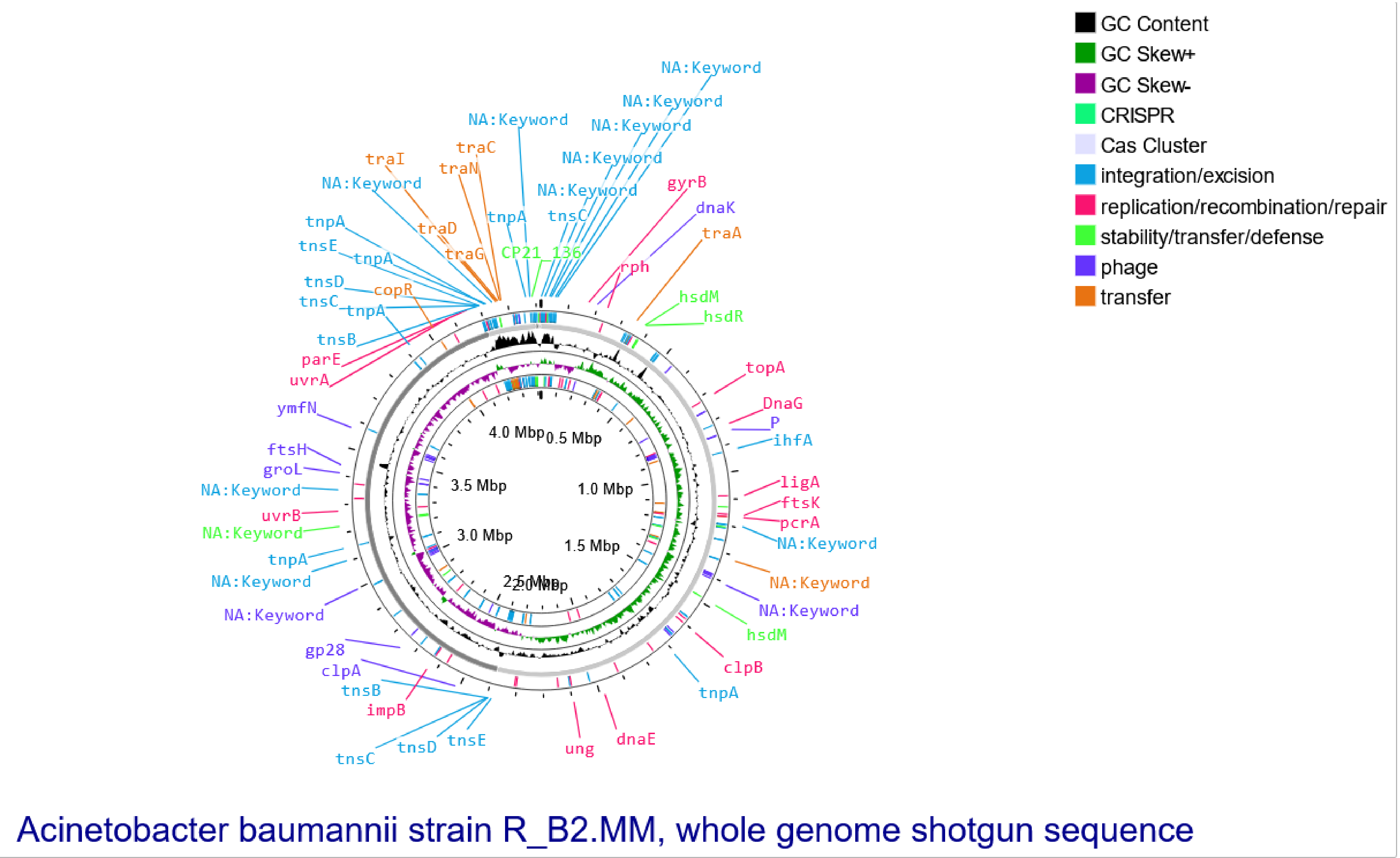

Figure S14. Mobile genetic elements found in the genome of A. baumannii R_B2.MM

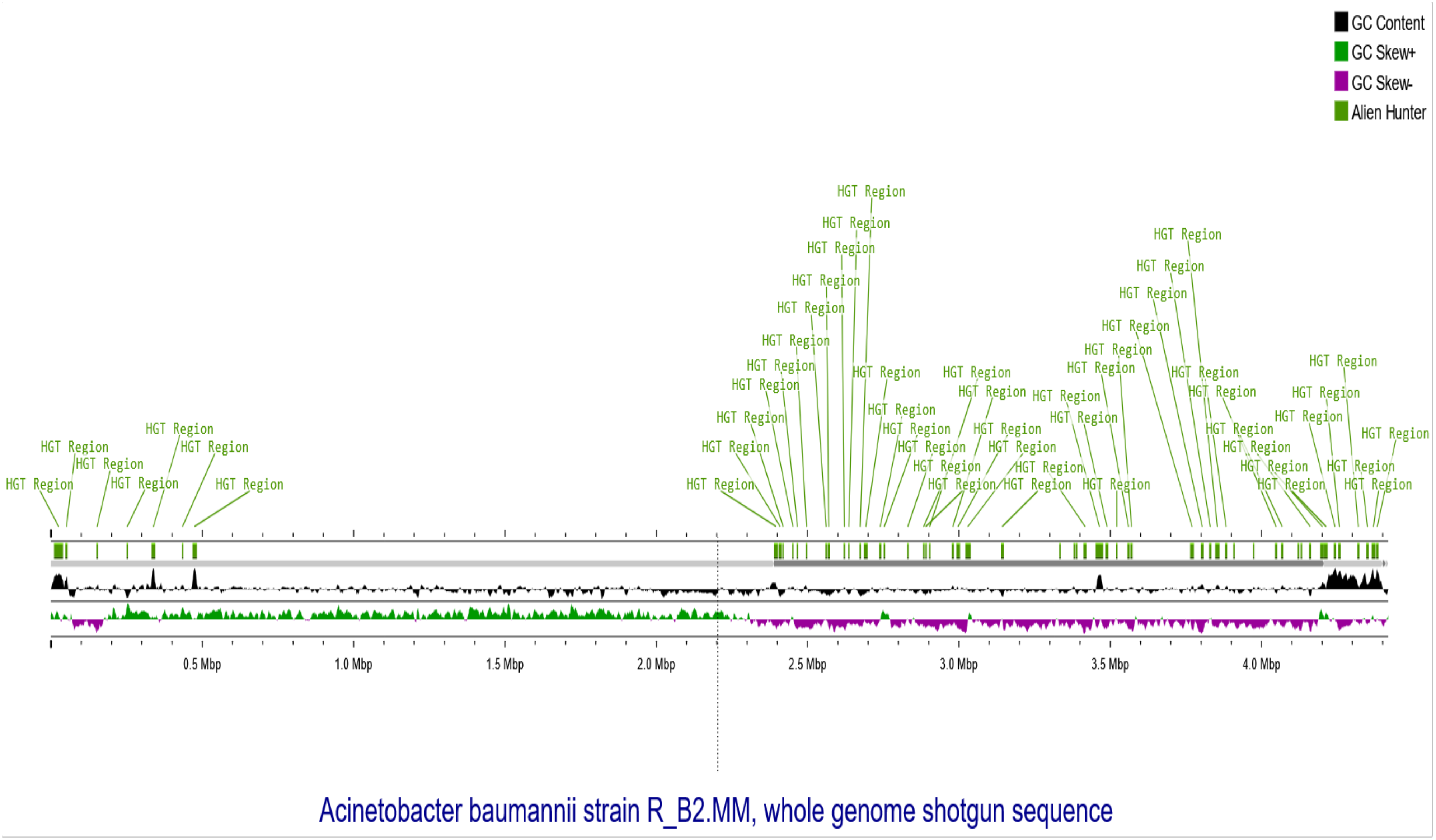

Figure S15. Regions of the A. baumannii R_B2.MM genome with active horizontal gene expression (HGT) events are annotated on the genome map

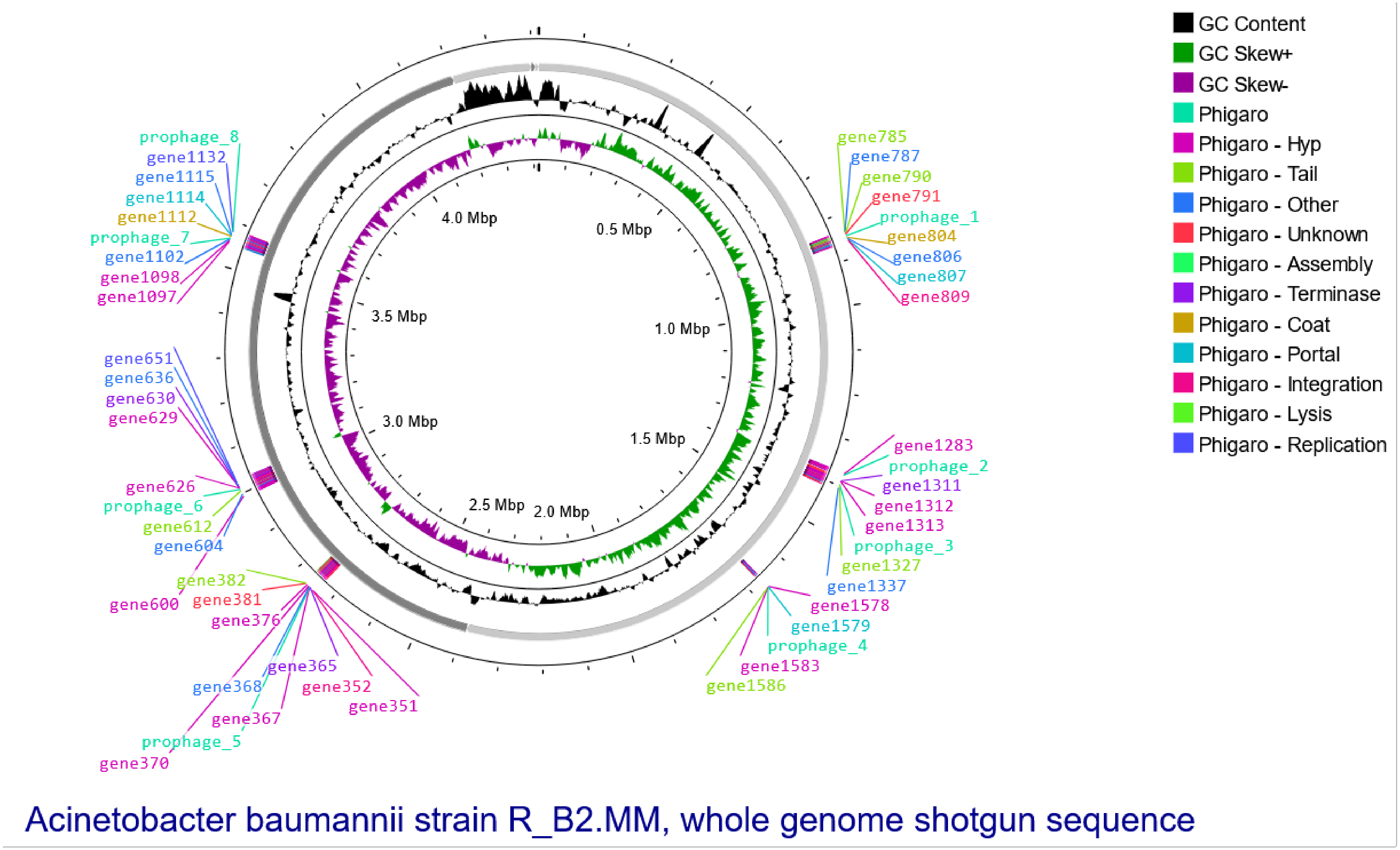

Figure S16. Types and distribution of prophages found in the A. baumannii R_B2.MM genome
